# Supplementary material for: High-fidelity musculoskeletal modeling reveals that motor planning variability contributes to the speed-accuracy tradeoff
Source: eLife. 2020 Dec 16;9:e57021. doi: 10.7554/eLife.57021 (PMC7787661; doi:10.7554/eLife.57021)
Supplement: Supplementary file 1. — For five subjects, we present the mean, standard deviation, minimum, and maximum of the movement durations in 10 reaches to a large (L) and small (S) square targets (see the section Fast reaching task). The target widths are 8 cm and 2 cm, and the reach amplitudes are 15 cm. The data are available at https://simtk.org/projects/ue-reaching/. [file elife-57021-supp1.docx]

| Subject | L mean(std) | L (min, max) | S mean(std) | S (min,max) |
| --- | --- | --- | --- | --- |
| 1 | 0.19(0.03) | (0.16,0.24) | 0.29(0.13) | (0.18,0.59) |
| 2 | 0.25(0.06) | (0.11,0.31) | 0.27(0.11) | (0.12,0.38) |
| 3 | 0.18(0.04) | (0.10,0.24) | 0.25(0.06) | (0.11,0.31) |
| 4 | 0.26(0.04) | (0.19,0.34) | 0.32(0.03) | (0.27,0.37) |
| 5 | 0.21(0.03) | (0.14,0.26) | 0.27(0.03) | (0.21,0.34) |

**Supplementary File 1. Table 1. Fast Reaching Experimental data**. For five subjects, we present the mean, standard deviation, minimum and maximum of the movement durations in ten reaches to a large (L) and small (S) square target (see *Fast Reaching Task*). The target widths are 8 cm and 2 cm, and the reach amplitudes are 15 cm. The data are available at https://simtk.org/projects/ue-reaching/.
